# Prevalence of common mental disorders among Syrian refugee children and adolescents in Sultanbeyli district, Istanbul: results of a population-based survey

**DOI:** 10.1017/S2045796020001079

**Published:** 2020-12-10

**Authors:** N. Scherer, S. Hameed, C. Acarturk, G. Deniz, A. Sheikhani, S. Volkan, A. Örücü, I. Pivato, İ. Akıncı, A. Patterson, S. Polack

**Affiliations:** 1 Department of Clinical Research, International Centre for Evidence in Disability, London School of Hygiene & Tropical Medicine, London, UK; 2 Department of Psychology, Koç University, Istanbul, Turkey; 3 Mülteciler Derneği, Istanbul, Turkey; 4 Relief International, Istanbul, Turkey

**Keywords:** Child psychiatry, Common mental disorders, Mental health, Population survey

## Abstract

**Aims:**

Research demonstrates elevated levels of common mental disorders among Syrian refugees, but the majority of studies have, to date, focused on adult populations. This study aims to estimate the prevalence of depression, anxiety and post-traumatic stress disorder (PTSD) among Syrian children and adolescents living in Sultanbeyli district of Istanbul, Turkey.

**Methods:**

A population-based survey among Syrian children and adolescents aged 8–17 years living in Sultanbeyli district was conducted in 2019, as part of an all-age survey of disability. 80 clusters of 50 participants (all-ages) were selected from the local municipality's refugee registration database using probability proportionate to size sampling. Children aged 8–17 years were assessed for symptoms of common mental disorders using the Child Revised Impact of Event Scale (CRIES-8) and abbreviated versions of the Center for Epidemiologic Studies Depression Scale for Children (CES-DC) and the Screen for Child Anxiety Related Disorders (SCARED).

**Results:**

Of the 852 participants, 23.7% (95% CI 19.9-27.2) screened positive for symptomatic depression, PTSD and anxiety. The prevalence estimates for depression, PTSD and anxiety were 12.5% (95% CI 9.8–15.6), 11.5% (95% CI 9.1–14.4) and 9.2% (95% CI 6.8–12.1), respectively. Depression and PTSD were significantly more common in older adolescents, whilst anxiety and PTSD were significantly more common in girls. Depression was more common in children from poorer households and those who had received no education. Children coming from larger households were less likely to show symptoms of PTSD.

**Conclusions:**

Syrian refugee children and adolescents are vulnerable to common mental disorders, and culturally appropriate prevention and intervention support are needed for this population.

## Introduction

Exposure to potentially traumatic events, post-migration challenges and other psychological stressors can place conflict-affected populations at an increased risk of common mental disorders, such as depression, anxiety and post-traumatic stress disorder (PTSD) (Fazel *et al*., 2005; Turrini *et al*., 2017; Giacco *et al*., 2018; Kien *et al*., 2019). Among displaced and conflict-affected populations, the World Health Organization (WHO) estimates the age-standardised prevalence of depression to be 10.8%, anxiety disorders (including PTSD) 21.7% and PTSD alone 15.3% (Charlson *et al*., 2019). These estimates are considerably higher than WHO estimates for common mental disorders among the general global population, at 4.4% for depression and 3.6% for anxiety disorders (including PTSD) (World Health Organization, 2017).

Included among those at risk are persons affected by the war in Syria, with an estimated 13.1 million requiring humanitarian assistance within Syria itself, and a further 5.6 million displaced across Europe and neighbouring countries (United Nations High Commissioner for Refugees, 2019). Evidence from these settings signals elevated levels of common mental disorders among displaced Syrian refugees. The majority of the available evidence comes from adult populations, with prevalence estimates ranging from 14% to 44% for depression (Naja *et al*., 2016; Tinghög *et al*., 2017; Acarturk *et al*., 2018; Georgiadou *et al*., 2018; Fuhr *et al*., 2019), 13% to 36% for anxiety (Tinghög *et al*., 2017; Georgiadou *et al*., 2018; Fuhr *et al*., 2019) and 11% to 83% for PTSD (Alpak *et al*., 2015; Tinghög *et al*., 2017; Acarturk *et al*., 2018; Chung *et al*., 2018; Georgiadou *et al*., 2018; Fuhr *et al*., 2019). Evidence among Syrian children is more limited, although findings consistently demonstrate symptoms of common mental disorders (Hamdan-Mansour *et al*., 2017; Yayan *et al*., 2020). The majority of these studies focus on PTSD, with prevalence estimates ranging from 33% to 50% (Ozer *et al*., 2013; Soykoek *et al*., 2017; Eruyar *et al*., 2018).

An estimated 3.6 million displaced Syrians live in Turkey, of whom almost half are children and adolescents under the age of 18 (United Nations High Commissioner for Refugees, 2020). One study, conducted in Islahiye camp near the Syria border, found that exposure to traumatic events and common mental disorders was high among Syrian children (Ozer *et al*., 2013). The majority (74%) had experienced a family member dying, and 58% had experienced a stressful life event in which they believed their life was in danger. Nearly half of children in this study were found to have symptoms of PTSD, and 20% were identified as having a depressive disorder (Ozer *et al*., 2013). Of these, significantly more girls than boys reported symptoms of depression.

The majority of Syrians (96%) in Turkey do not live in refugee camps, but rather live among the host population. This includes 550 000 Syrians currently living in Istanbul (Ministry of Interior: Directorate General of Migration Management, 2019). A survey among adult Syrian refugees living in Sultanbeyli district of Istanbul, conducted in 2018, found a high prevalence of depression (34.7%), anxiety (36.1%) and PTSD (19.6%), with the majority screening positive for these conditions not seeking care of any kind. However, data were not collected for children (Fuhr *et al*., 2019). To the best of our knowledge, just one study has previously been conducted with Syrian children living among the host population in Istanbul, with common mental disorders found to be common (Eruyar *et al*., 2018). Half of the children surveyed scored above the clinical cut-off for PTSD, with 24% judged to have ‘very high’ general mental health problems. However, this was a school-based study and community-based surveys are lacking.

Epidemiological data, identifying prevalence and factors associated with increased risk of common mental disorders, are important for informing the development of public mental health policy, special education and psychosocial support initiatives, and programming for Syrian children living among the host population in Turkey.

The objectives of the current study are to: (1) estimate the prevalence of symptoms of common mental disorders (depression, anxiety and PTSD) among Syrian refugee children living in Sultanbeyli district of Istanbul; and (2) identify associations between symptoms of common mental disorders and socio-demographic characteristics.

## Methods

### Study design and sampling

This cross-sectional study was conducted between August and October 2019 in Sultanbeyli District, Istanbul, as part of an all-age population-based survey of disability among Syrian refugees, aged 2+ years. This paper reports specifically on mental health data collected among children and adolescents aged 8–17 years.

Based on evidence from similar populations, the prevalence of common mental disorders among children and adolescents aged 8–17 years was estimated to be 15%. Incorporating precision of 20% around the estimates, 95% confidence, 20% non-response and a design effect of 1.7, we required a sample size of 1073 participants aged 8–17 years. The total sample size calculated for the all-age survey of disability was 4000.

The local municipality's database of registered Syrian refugees provided the sampling frame, from which we selected the study population using a two-stage sampling technique. First, we randomly selected 80 clusters, of 50 participants each (a total all-age sample of 4000), using probability proportionate to size sampling. A cluster was defined as a single street within Sultanbeyli District. Within each cluster, we randomly selected households until at least 50 participants were included. When a single street (‘cluster’) did not contain 50 participants, we randomly selected additional households from randomly selected connecting and adjacent streets, until the target number was reached.

Across all 80 clusters, enumerators visited households door-to-door, tallying participants until at least 50 people were included. For this survey, all Syrians within selected households were included in the survey, regardless of their legal ‘Temporary Protection’ status. To maximise the response rate, enumerators telephoned households in advance, where possible, to inform them of the survey purpose and arrange a suitable time to visit. If unavailable, enumerators attempted to revisit the household at another time, including evenings and weekends.

### Survey questionnaires

Upon consent from the head of each household, enumerators completed a household roster, gathering socio-demographic information.

Symptoms of common mental disorders were subsequently assessed in each child aged 8–17 years using direct self-report questionnaires. Depression was assessed using the Center for Epidemiologic Studies Depression Scale for Children (CES-DC), anxiety using the Screen for Child Anxiety Related Disorders (SCARED) and PTSD using the Child Revised Impact of Event Scale (CRIES-8). Each of these tools has been previously used in research with refugee populations (Eruyar *et al*., 2018; Kandemir *et al*., 2018; Perkins *et al*., 2018). To limit the response burden, we used abbreviated versions of the CES-DC and SCARED, validated for use with Syrian refugee children living in Lebanon (Mcewen *et al*., 2020).

Each of these screening tools results in a total score, indicating high or low symptomology. These tools are designed to screen symptomology, not to provide a diagnosis, which is typically done through a clinical interview with a trained mental health specialist. Screening tools of this kind have typically been validated against clinical interviews and the cut-off scores indicate the level at which a child is likely to receive a diagnosis.

#### Center for Epidemiologic Studies Depression Scale for Children (CES-DC)

The CES-DC measures the frequency and duration of symptoms associated with depression in children and adolescents (Faulstich *et al*., 1986). This measure has been adapted to an abbreviated 10-item version (CES-DC-10Sy) by Queen Mary University and their research partners, for use with Syrian children in Lebanon (Mcewen *et al*., 2020). The 10-item version showed good internal consistency (Cronbach's *α* = 0.89) and was used in this study after consultation with the research team at Queen Mary University. This abbreviated version asks children to respond to statements (‘items’) across a four-point Likert scale, identifying to what extent they experienced a given feeling in the past month: (0) not at all; (1) a little; (2) some; (3) a lot. The validated cut-off score among Syrian children of 10, from a total of 30, indicates symptomatic depression and those children at risk of clinical diagnosis (Mcewen *et al*., 2020).

#### Screen for Child Anxiety Related Disorders (SCARED)

The SCARED screens children and adolescents for anxiety disorders, including general anxiety disorder, panic disorder and separation anxiety disorder (Birmaher *et al*., 1997). We used the abbreviated version developed by Queen Mary University (SCARED-18Sy), previously validated for use with Syrian children in Lebanon, and found to have good internal consistency (Cronbach's *α* = 0.84) (Mcewen *et al*., 2020). The abbreviated measure used in this study scored the 18 items across a three-point scale: (0) not true or hardly ever true; (1) somewhat true or sometimes true; (2) very true or often true. The validated cut-off of 12, from a maximum score of 36, indicates symptomatic anxiety (Mcewen *et al*., 2020).

#### Child Revised Impact of Event Scale (CRIES-8)

The CRIES tool is designed to assess symptoms of PTSD (Perrin *et al*., 2005). The eight-item version (hence CRIES-8) demonstrates good psychometric properties among conflict-affected populations and has been previously used with Syrian refugee children in Turkey (Ozer *et al*., 2013; Eruyar *et al*., 2018). Among refugee minors in Sweden (including Arabic speakers), internal consistency was found to be acceptable (Cronbach's *α* = 0.75) (Salari *et al*., 2017). The tool is designed to measure the intrusion and avoidance of unwanted thoughts, feelings and memories, and items are scored on a four-point scale: (0) not at all; (1) rarely; (3) sometimes; (5) often. Items ask children to report how frequently comments were true for them in the past 7 days, when thinking about a stressful life event. From a maximum score of 40, the cut-off of 17 indicates symptomatic PTSD.

#### Child Youth and Resilience Measure (CYRM)

In addition to an assessment of symptoms of common mental disorders, we also assessed resilience in children aged 12–17 years, using a modified 12-item version of the CYRM, which has been validated for use with Syrian adolescents (Panter-Brick *et al*., 2018). Internal consistency was found acceptable (Cronbach's *α* = 0.75) in this validation study. Using this tool, children are asked to rate the extent to which a statement is relevant to them, on a four-point scale: (1) not at all; (2) a little; (3) somewhat; (4) quite a bit. Higher scores indicate a higher level of personal resilience as a protective factor against the onset of mental distress, with a maximum score of 48.

Existing Arabic versions of each tool were independently back-translated into English, assessing accuracy, conceptual equivalence and cultural acceptance. These tools were subsequently piloted with members of the target population by technical experts (each a native Syrian Arabic speaker), who shared recommendations of minor amendments for use with this population.

### Data collection

Enumerators administered each of the self-report questionnaires directly with a child, within their home. If requested (by child or caregiver), a parent/guardian/caregiver remained present throughout the interview. Enumerators conducted the interview in Arabic. Visual aids (an example of which is seen in Appendix 1) were used to help children understand the questionnaire responses. Each of the enumerators had completed a 10-day training, covering the aims of the survey, interview techniques, mental health and disability sensitisation, data entry procedures and ethical responsibilities.

Data were collected on android tablets using the London School of Hygiene & Tropical Medicine's Open Data Kit software. Collected data were encrypted and uploaded to a secure cloud-based server at the end of each day.

### Data analysis

Data were analysed using STATA version 14.0. Prevalence estimates (with 95% CI) were calculated from those children scoring at or above the cut-off on each measurement tool. These estimates were stratified by age, sex and socio-economic status. The ‘svv’ command in STATA was used to account for the cluster sampling methods.

Principal components analysis was used to derive a socio-economic index from household level indicators such as household asset ownership, type of residence and source of heating. The resulting socio-economic score was divided into quartiles, from the poorest to least poor. The first principal component accounted for 14.96% of the variation in the original data.

Multivariate regression analyses were undertaken to assess the relationship between each common mental disorder and individual (age, sex, years since displacement, education and resilience), household level (household size, household head and socio-economic status) and socio-demographic characteristics.

### Ethical approval

Ethical approval was granted by Istanbul Sehir University, Republic of Turkey Ministry of Interior: Directorate General of Migration Management and the London School of Hygiene & Tropical Medicine.

Informed written consent was sought from the self-identified head of each household prior to completion of the household roster and collection of demographic information. Informed consent was subsequently sought from parents and caregivers for children and adolescents aged 8–17 years. Verbal assent was also sought from the children and adolescents.

## Results

### Study population

Of 1080 eligible participants aged 8–17 years, 852 took part in the survey (response rate of 79%), 157 were unavailable (15%) and 71 (7%) refused. The sample characteristics of these participants are provided in Table 1.

**Table 1.** Sample characteristics (*n* = 852)

	Total	
	*n*	%
Age (years)		
8–10	322	38
11–13	273	32
14–17	257	30
Mean (95% CI)	11.9 (11.7–12.1)	
Sex		
Male	413	49
Female	439	51
Years since leaving Syria^a^		
<2 years	54	6
2–3 years	155	18
4–5 years	476	56
6–8 years	164	19
Mean (95% CI)	4.3 (4.2–4.4)	
Highest level of education^a^		
Never attended	54	6
Primary	621	73
Middle/Secondary	171	20
Accommodation type^a^		
Flat/apartment	683	81
House	136	16
Basement	19	2
Store/warehouse	10	1

a Data missing.

Of the study sample, 439 (51%) were female and 413 (49%) male. Compared to participants, non-participants were, on average, slightly older (participants: 11.9 years, 95% CI 11.7–12.9 years; non-participants: 12.9 years, 95% CI 12.5–13.2, *p* < 0.01) and more likely to be male (participants: 49%; non-participants: 59%). Overall, the sample age distribution was largely comparable to the registration database used for the sampling frame, with slight under-representation of older children.

On average, participants in the study had been displaced from Syria approximately 4 years prior to the study. The vast majority of children lived in a flat/apartment (81%) or house (16%). In terms of the highest education level attained, 73% had attended primary school, 20% had attended secondary school and 6% had never attended formal education (given the age distribution of the sample, it is important to note that not all children were eligible to have attended secondary school).

### Prevalence of common mental disorders

The estimated prevalence of symptomatic depression, anxiety and PTSD were 12.5% (95% CI 9.8–15.6), 9.2% (95% CI 6.8–12.1) and 11.5% (95% CI 9.1–14.4), respectively (Table 2). Overall, 23.7% (95% CI 19.9–27.2) had symptoms of one or more of these common mental disorders.

**Table 2.** Prevalence of common mental disorders, by age and sex

	Depression % (95% CI)	Anxiety % (95% CI)	PTSD % (95% CI)
Overall	12.5% (9.8–15.6)	9.2% (6.8–12.1)	11.5% (9.1–14.4)
Age (years)			
8–10	6.8% (4.3–10.6)	8.4% (5.6–12.3)	7.1% (4.8–10.6)
11–13	11.7% (8.1–16.6)	9.5% (6.1–14.7)	10.3% (6.9–14.9)
14–17	20.6% (14.9–27.7)^±^	9.7% (6.3–14.5)	18.3% (13.2–24.7)^±^
Sex			
Male	11.1% (8.0–15.2)	4.8% (3.1–7.3)	9.2% (6.7–12.5)
Female	13.9% (10.3–18.3)	13.2% (9.5–18.0)^±^	13.7% (10.4–17.7)^±^

± Significant at *p* < 0.05.

As seen in Table 3, 16.4% screened positive for symptoms of just one condition only, 5.3% showed symptoms across two conditions and 2.1% were found to have symptoms of all three common mental disorders.

**Table 3.** Distribution of symptomology, by common mental disorder

	Male (*n* = 413)		Female (*n* = 439)		Total (*n* = 852)	
	*n*	%	*n*	%	*n*	%
Scoring below clinical cut-offs	334	80.9	316	72.0	650	76.3
Symptoms of one condition						
Depression only	26	6.3	29	6.6	55	6.5
Anxiety only	9	2.2	25	5.7	34	4.0
PTSD only	23	5.6	27	6.2	50	5.9
Symptoms of two conditions						
Depression and anxiety	6	1.5	9	2.1	15	1.8
Depression and PTSD	10	2.4	9	2.1	19	2.2
PTSD and anxiety	1	0.2	10	2.3	11	1.3
Symptoms of three conditions						
Depression, anxiety and PTSD	4	1.0	14	3.2	18	2.1

### Association with resilience

Children with symptoms of depression demonstrated significantly lower mean resilience scores (59.0, 95% CI 55.0–63.1) compared to children without symptomatic depression (71.2, 95% CI 69.5–72.9), as shown in Table 4. There was no association observed between resilience score and symptoms of anxiety or PTSD.

**Table 4.** Association between common mental disorders and resilience

	Mean (95% CI)^a^	*p*-value (adjusted for age and sex)
Symptomatic depression		
Without	71.2 (69.5–72.9)	<0.001
With	59.0 (55.0–63.1)	
Symptomatic anxiety		
Without	68.9 (67.1–70.7)	0.81
With	69.7 (65.3–74.1)	
Symptomatic PTSD		
Without	69.1 (67.2–79.9)	0.53
With	69.1 (65.6–72.6)	

a Higher score indicates a greater level of resilience.

### Association with socio-demographic variables

Table 5 presents the association between each common mental disorder and socio-demographic variables, adjusted for age and sex. Older children (14–17 years) were three times more likely to experience symptomatic depression (adjusted odds ratio (aOR): 3.6, 95% CI 2.1–6.8) and PTSD (aOR: 3.0, 95% CI 1.7–5.2) compared to younger (8–10 years) children, although this association was not observed for anxiety. Symptomatic anxiety (aOR: 2.9, 95% CI 1.8–4.9) and PTSD (aOR: 1.6, 95% CI 1.1–2.4) were more common among girls, with no association observed for depression. Demonstrating symptoms of these common mental disorders was not significantly associated with the number of years since leaving Syria. Symptomatic depression was more common in children who had received no education (aOR: 2.3, 95% CI 1.0–5.2), although no association was seen for PTSD or anxiety. Symptomatic PTSD was found to be significantly less likely to occur in children living in a household with 8+ members (aOR: 0.5, 95% CI 0.3–0.9), compared to those living in households of 2–4. Household size was not associated with symptomology of depression or anxiety, and the sex of the head of the household was not associated with any common mental disorder. Symptomatic depression was slightly more common among children who lived in the poorest households, although this was of borderline significance (aOR: 2.0, 95% CI 1.0–3.5). Socio-economic status was not associated with either PTSD or anxiety.

**Table 5.** Association between common mental disorders and socio-demographic variables

	Children without depression		Children with depression		Age and sex adjusted OR (95% CI)	Children without anxiety		Children with anxiety		Age and sex adjusted OR (95% CI)	Children without PTSD		Children with PTSD		Age and sex adjusted OR (95% CI)
	*n*	%	*n*	%		*n*	%	*n*	%		*n*	%	*n*	%	
Age (years)															
8–10	300	40	22	20	Reference	295	38	27	35	Reference	299	40	23	23	Reference
11–13	241	32	32	30	1.8 (1.1–3.0)^±^	247	32	26	33	1.1 (0.6–2.1)	245	32	28	29	1.5 (0.8–2.7)
14–17	204	27	53	50	3.6 (2.1–6.8)^±^	232	30	25	32	1.2 (0.7–2.0)	210	28	47	48	3.0 (1.7–5.2)^±^
Sex															
Male	367	49	46	43	Reference	393	51	20	26	Reference	375	50	38	39	Reference
Female	378	51	61	57	1.3 (0.8–2.0)	381	49	58	74	2.9 (1.8–4.9)^±^	379	50	60	61	1.6 (1.1–2.4)^±^
Years since leaving Syria^a^															
<2 years	43	6	11	9	1.6 (0.5–2.8)	47	6	5	7	1.2 (0.4–3.4)	44	6	8	8	1.7 (0.6–4.8)
2–3 years	139	19	16	15	0.7 (0.3–1.5)	142	18	13	18	1.6 (0.8–3.2)	136	18	22	22	1.5 (0.8–3.0)
4–5 years	419	56	57	53	0.8 (0.4–1.4)	430	56	44	60	1.4 (0.7–2.6)	423	56	52	53	1.1 (0.6–2.2)
>5 years	141	19	23	22	Reference	152	20	12	16	Reference	148	20	16	16	Reference
Highest level of education^a^															
None	43	6	11	11	2.3 (1.0–5.2)^±^	48	6	6	8	0.9 (0.3–2.6)	46	6	8	8	1.0 (0.5–2.2)
Primary	549	74	72	69	1.4 (0.8–2.3)	573	74	48	64	0.6 (0.3–1.0)	560	74	61	62	0.7 (0.4–1.3)
Middle/secondary	149	20	22	21	Reference	150	20	21	28	Reference	142	19	29	30	Reference
Household level variables															
Household size															
2–4 people	97	13	14	13	Reference	105	14	9	12	Reference	96	13	18	18	Reference
5–7 people	454	61	73	68	1.0 (0.5–2.0)	476	62	50	64	1.1 (0.5–2.5)	469	62	57	58	0.6 (0.3–1.0)
8+ people	194	26	20	19	0.6 (0.2–1.3)	193	25	19	24	1.0 (0.5–2.0)	189	25	23	23	0.5 (0.3–0.9)^±^
Sex of household head															
Male	579	78	78	73	Reference	599	77	58	75	Reference	590	78	67	68	Reference
Female	166	22	29	27	1.2 (0.7–2.0)	175	23	20	25	1.1 (0.6–2.1)	164	22	31	32	1.5 (0.9–2.6)
Socio-economic status^a^															
First quartile (poorest)	179	24	38	36	2.0 (1.0–3.5)	198	26	19	26	1.0 (0.5–1.9)	197	26	20	20	0.7 (0.4–1.4)
Second quartile	184	25	31	29	1.5 (0.9–2.6)	195	25	21	28	1.1 (0.5–2.3)	194	26	22	22	0.8 (0.4–1.4)
Third quartile	200	27	18	17	0.8 (0.4–1.7)	199	26	19	25	0.9 (0.4–1.9)	185	25	33	34	1.3 (0.7–2.3)
Fourth quartile (least poor)	177	24	18	17	Reference	179	23	16	21	Reference	172	23	23	23	Reference

± Significant at *p* < 0.05.

a Data missing.

## Discussion

This study reports on the prevalence and socio-demographic predictors of common mental disorders among Syrian children living in Istanbul. Nearly a quarter (23.7%) of children screened positive for symptoms of at least one common mental disorder. By condition, 12.5% screened positive for symptomatic depression, 9.2% for anxiety and 11.5% PTSD. Older children were more likely to screen positive for symptomatic depression and PTSD, with girls more likely to screen positive for anxiety and PTSD. Depression was more common among children who had received no education and were from the poorest households.

These estimates fall at the lower end of prevalence figures identified in a recent systematic review of studies among young refugees (under 18 years) displaced across Europe, in which estimates ranged from 10.3–32.8% for depression, 19–52.7% for PTSD and 8.7–31.6% for anxiety (Kien *et al*., 2019). Our findings on PTSD do, however, align with evidence from an umbrella review conducted by Turrini *et al*., which identified five surveys of 260 refugee children that generated an estimate of 11% for PTSD, similar to that in our study (Turrini *et al*., 2017). The wide variation in these estimates may reflect the varying study and sampling methodologies, and the different screening tools, as well as the different characteristics of the refugee groups and host communities.

Overall, our study indicates a substantial burden of common mental disorders among Syrian children in Istanbul, with nearly a quarter of children demonstrating symptoms of at least one common mental disorder. Our estimates are considerably higher than estimates from the WHO for the general population and are congruent with the evidence of a substantial burden of common mental disorders among Syrian refugees (Ozer *et al*., 2013; World Health Organization, 2017; Fuhr *et al*., 2019).

Eruyar *et al*.'s study among Syrian school children in Istanbul estimated the prevalence of PTSD to be 50% using CRIES-8, the same screening tool used in our study (Eruyar *et al*., 2018). This estimate is considerably higher than that identified in our study, although their sample was recruited from two Syrian schools only, limiting the comparison to our community-based survey. Similarly, the prevalence of PTSD was estimated to be higher among children living in Islahiye camp, in South-Eastern Turkey (Ozer *et al*., 2013). This study also utilised the CRIES-8, with 45% of children reaching the cut-off for symptomatic PTSD. Further, 44% screened positive for depression, of whom 20% reached the clinical cut-off, albeit via a different screening tool to that used in our study. Comparison between these estimates and ours may reflect a greater risk of common mental disorders among those refugees living in a camp setting, compared to those living in an urban setting among the host population, as with our sample. Additionally, the study in Islahiye camp was conducted in 2012, just one year after the start of the war in Syria, and the study in Syrian schools was conducted in 2015. In contrast, 75% of children in our 2019 study had been away from Syria for at least 4 years, and the difference in PTSD estimates may indicate that children with more recent exposure to the war and associated trauma are at a higher risk of symptoms of PTSD.

Our study identified a number of risk and protective factors associated with common mental disorders among Syrian children in Istanbul. Children with symptomatic depression in our sample were more likely to come from poorer households, consistent with evidence on the social determinants of mental health among the general and refugee populations (Porter and Haslam, 2005; Allen *et al*., 2014; Hynie, 2018).

Symptoms of depression were also more common in children who had not attended school. School is crucial to a child's integration and acculturation in the host community, providing children with a place in which to develop relationships and a sense of belonging. Evidence indicates that the social support structures provided by a school offer protection against common mental disorders (Fazel and Stein, 2002), although the mechanism of this among Syrian children in Istanbul merits further investigation.

Similar structures may explain our finding that children from households with a larger number of household members were significantly less likely to demonstrate symptoms of PTSD. Social support is a strong protective factor for common mental disorders (Marley and Mauki, 2018), and children from larger households may receive more stable social support, shielding them against symptoms of PTSD. This is consistent with the ‘Intervention Pyramid’ proposed in the *Inter-Agency Standing Committee Guidelines on Mental Health and Psychosocial Support in Emergency Settings*, which highlights the need for strengthened community and family supports for those affected by situations of emergency, displacement and conflict (Inter-Agency Standing Committee, 2007).

As was expected, symptomatic depression was inversely associated with resilience scores, consistent with Panter-Brick *et al*.'s study among Syrian youth (Panter-Brick *et al*., 2018). This association was not, however, observed for symptoms of anxiety or PTSD, despite support from the literature on the role of resilience as a protective factor against the onset of these disorders (Charney, 2003). Further exploration is required in the interaction of resilience with social factors among Syrian children living in Istanbul.

Important to consider among our findings is the higher prevalence of common mental disorders in girls (27.7%) compared to boys (18.8%). This is consistent with research from across refugee and general populations, in which girls are reported to have a higher prevalence of depression and suicidal ideation/attempts than boys (Afifi, 2007; Mohwinkel *et al*., 2018). Gender differences in anxiety and PTSD are less well defined, but apparent in some studies (Van Droogenbroeck *et al*., 2018). This includes our study, in which girls (13.2%) demonstrated a significantly higher prevalence of symptomatic anxiety compared to boys (4.8%).

Evidence suggests that girls are more likely to exhibit ‘internationalising’ behaviours (withdrawal, self-consciousness and hypersensitivity), as opposed to ‘externalising’ behaviours (aggression, non-compliance and impaired self-regulation) in boys, particularly in adolescence (Liu *et al*., 2011). Males and females also exhibit differences in help-seeking, coping strategies and preferences for treatment (Liddon *et al*., 2018). These are important considerations for future prevention and intervention strategies for this population.

### Limitations

This was the first population-based survey of common mental disorders in Syrian refugee children living among the host community in Istanbul. Survey teams visited participants in their households, which may have contributed to the higher response rate than a previous study of adult mental health in the same district (Fuhr *et al*., 2019).

However, there were some limitations. Firstly, the sample was selected from the Sultanbeyli municipality's refugee registration database, which does not include information of unregistered or undocumented refugees, who may have a higher prevalence of common mental disorders. We sought to address this to some extent by including all eligible Syrians within a household, regardless of legal refugee status, but this cannot account for those unregistered households who would have been missed during the first stages of sampling.

Second, the response rate was slightly lower than 80%, although age and sex distribution were largely congruent with the registration database.

Third, all efforts were made to interview participants in private, but this was not always possible, as interviews were conducted in the child's home and caregivers had the right to be present. This may have resulted in response bias, especially with the sensitive nature of questions on mental health. Arguably, considering the stigma often attached to mental health, this may have resulted in underestimates of common mental disorders. The number of participants interviewed in the presence of caregivers was not recorded, which would have aided further analysis.

Fourth, a clinical diagnostic interview would have resulted in more precise prevalence estimates, but this was not feasible within a population survey. Self-report mental health screening tools can result in overestimated prevalence figures, although this is an issue common to nearly all other studies (Thombs *et al*., 2018).

Finally, we used abbreviated versions of tools used to assess symptoms of depression and anxiety. The tools and cut-offs for these abbreviated versions have been validated with Syrian children living in refugee camps in Lebanon, as opposed to children living among a host population, as is the case in Sultanbeyli (Mcewen *et al*., 2020). These validated cut-offs may be higher than needed for children in Sultanbeyli, as children living in camps may be more likely to endorse certain items, given more challenging living conditions. As such, our estimates for symptomatic depression and anxiety in children may represent an under-estimate. Similarly, the tool used to assess symptoms of PTSD has been validated among Arabic speaking and refugee populations, but not among Syrians specifically.

## Conclusion

We have presented findings of the first population-based study to report on the estimated prevalence of symptomatic depression, anxiety and PTSD among Syrian children and adolescents living in Istanbul, Turkey. Nearly 25% of participants demonstrated symptoms of at least one common mental disorder. The estimates of this study are considerably higher than in the general population and represent a substantial burden of these common mental disorders in this population. With high estimates of common mental disorders among Syrian children in Istanbul, it is important to support mental health intervention and prevention policies and programmes for this group.

## Data Availability

For more information on the data supporting the findings of this study, please contact Nathaniel Scherer: nathaniel.scherer@lshtm.ac.uk
